# In Situ Differential Analysis of α- and β-Glycosidase Activities in Lysosomes After Internalization Using Glucosylcerebroside-Based Liposomes

**DOI:** 10.3390/ijms27062749

**Published:** 2026-03-18

**Authors:** Yi Wei, Osamu Kanie

**Affiliations:** 1 Graduate School of Science and Technology, Tokai University, Hiratsuka 259-1292, Kanagawa, Japan; 0mtad001@tokai.ac.jp; 2 Department of Bioengineering, Tokai University, Hiratsuka 259-1292, Kanagawa, Japan; 3 Micro/Nano Technology Center, Tokai University, Hiratsuka 259-1292, Kanagawa, Japan

**Keywords:** glucosylceramide (GlcCer), fluorogenic glycosides, 4-methylumbelliferone, 4-(trifluoromethyl)umbelliferone, differential analysis, confocal imaging, liposome

## Abstract

Fluorogenic glycosides are widely used substrates for assaying lysosomal glycosidase activities in vitro, but they do not provide subcellular information in living cells. In this study, we used glucosylceramide (GlcCer) liposomes as carriers to deliver fluorogenic substrates into live PC12 cells for confocal imaging. The α-4-methylumbelliferyl glucoside (α-4MUG) and β-glucosidase substrate β-4-(trifluoromethyl)umbelliferyl glucoside (β-4FMUG) were co-encapsulated in liposomes. The liposomes (approximately 100 nm in diameter) were taken up by PC12 cells after pulse exposure. Punctate fluorescence signals from both hydrolyzed substrates were observed. The relative intensity of two signals varied among puncta, as assessed by dual-channel imaging and line-scan analysis. These results show that GlcCer liposomes provide a practical platform for long-term and differential analyses of relative α- and β-glucosidase activities in living cells.

## 1. Introduction

Glycosidases in lysosomes and the endoplasmic reticulum (ER) are important for the turnover of glycosphingolipids/glycoconjugates and for quality control during protein glycan processing [1,2]. Aberrations in these pathways are closely associated with various lysosomal storage disorders (e.g., Gaucher and Pompe diseases) and related neurological pathologies. Among lysosomal glycosidases, glucocerebrosidase (GCase) and acid α-glucosidase (GAA) are clinically important [3,4,5], and their activities are commonly assessed using the corresponding fluorogenic substrates 4-methylumbelliferyl α/β-D-glucopyranosides (4MUGs), which release 4-methylumbelliferone (4MU) upon hydrolysis [6,7]. The hydrolytically released 4MU exhibits characteristic excitation/emission wavelengths ideal for fluorescence detection [8]. However, conventional fluorescence assays are usually performed using lysed samples or in vitro systems, which fail to provide direct information on the subcellular localization and spatiotemporal dynamics of enzymatic activity within live cells [9]. Therefore, the development of strategies for in situ readouts inside living cells remains crucial.

Liposomes and other lipid-based nanocarriers present an attractive platform for delivering hydrophilic, membrane-impermeable fluorescent substrates [10]. Encapsulating substrates within the aqueous lumen of liposomes minimizes their exposure to the extracellular environment and offers the potential to modulate their release and subsequent reaction processes following cellular uptake. Among lipid components, glucosylceramide (GlcCer) is a structurally simple yet critically important molecule that occupies the starting point of complex glycosphingolipid biosynthesis and is intimately linked with both health and disease states [11]. Previous studies have reported that liposomes composed mainly of GlcCer are internalized upon exposure to cells and can significantly influence membrane properties, such as fluidity [12]. Furthermore, research using a three-dimensional cultured skin epidermis model demonstrated that GlcCer-based liposomes, upon uptake, can elevate cellular ceramide levels, indicating their potential as bioactive delivery systems that interact with lipid metabolism, beyond merely serving as carriers of encapsulated molecules [13].

In the present work, we used PC12 cells primarily as a convenient model for intracellular imaging of glycosidase activity, noting that PC12 cells have also been employed in studies of glycosphingolipid metabolism [14]. Prior research has shown dynamic regulation of glycosphingolipid composition and related metabolism, including GlcCer synthesis/glycosphingolipid homeostasis during PC12 differentiation [15,16], supporting the use of this model to investigate interactions between glycosphingolipids and neural function. Additionally, PC12 cells have been utilized in studies involving the liposome-mediated delivery of membrane-impermeable molecules and the subsequent induction of cellular responses [17], establishing their suitability as a model for liposome-based intracellular delivery and imaging applications.

GlcCer has been incorporated into phospholipid membranes to modulate liposomal rigidity and robustness, and tuning the GlcCer fraction can alter membrane properties [18]. GlcCer-based liposomes have also been applied in biological models, supporting their potential as biobased lipid carriers [13].

**Figure 1:**
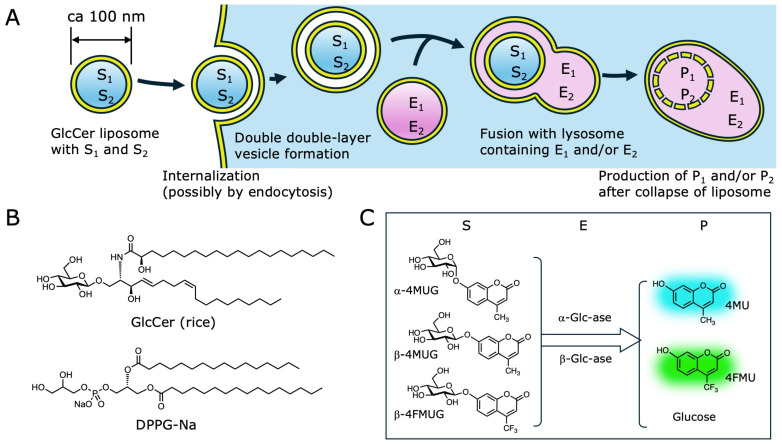
Schematic design and characterization of GlcCer-based liposomes encapsulating fluorogenic glycosidase substrates. (**A**) Schematic of the imaging concept. A GlcCer liposome carries two substrates (S_1_ and S_2_). The scheme depicts cellular uptake by endocytosis, formation of a vesicle with a double lipid bilayer, fusion with a lysosome containing enzymes (E_1_ and/or E_2_), and generation of products (P_1_ and/or P_2_) after liposome disruption. (**B**) Chemical structures of rice-derived GlcCer and DPPG-Na used for liposome preparation. (**C**) Expected glucosidase reactions inside lysosomes and structures of substrates and products. α-4MUG and β-4MUG yield 4MU (sky blue) after α-/β-glucosidase actions. β-4FMUG yields 4FMU (green) after hydrolysis by β-glucosidase.

In this study, we constructed a liposomal system utilizing rice bran-derived GlcCer as the primary membrane lipid (GlcCer content > 95%) for the rapid delivery of fluorogenic glycosidase substrates into live PC12 cells with our expectation that tight membrane packing of GlcCer due to the neutral and hydrogen-bonding capability of the head group would help vesicles maintain integrity and minimize premature cargo leakage in the acidic and enzyme-rich lysosomal environment [18,19]. Given the higher phase-transition temperature of GlcCer [20], we expected slow drug release from liposomes containing GlcCer, which is crucial for drug delivery. This led us to focus on this aspect. Additionally, reports show that liposomes with GlcCer are quickly internalized by cultured cells [21]. We also found that liposomes with 95% GlcCer are rapidly taken up by cells and localize to lysosome-like structures [12]. In this study, we further examined the selective glycoside hydrolysis of fluorogenic substrates inside internalized liposomes. We targeted two lysosomal enzymes, α-Glc-ase [22,23] and β-Glc-ase [24,25], both associated with diseases. Our goals are (1) to verify if GlcCer liposomes move to lysosomes after cell uptake, and (2) if confirmed, to determine where these two enzymes are located within the lysosomes. Furthermore, incorporating glucosides onto the liposome surface may enable receptor-mediated endocytosis [26]. We selected the α- and β-anomers of 4-methylumbelliferyl glucopyranoside (α-4MUG and β-4MUG), whose enzymatic hydrolysis yields fluorogenic 4MU upon action of individual α- and β-glucosidases [27,28] (Figure 1). To enable simultaneous identification of these enzyme activities within the same cell, we decided to utilize a green-fluorescent fluorophore, 4-(trifluoromethyl)umbelliferone (4FMU), and used its β-glycoside of glucopyranose (hereafter β-4FMUG). The specific aims of this study were to: (1) characterize the cellular uptake of liposomes consisting mainly of GlcCer (GlcCer liposomes) in undifferentiated PC12 cells; (2) compare the temporal changes and spatial distribution patterns of the 4MU signal derived from α-4MUG, β-4MUG and β-4FMUG in live cells; and (3) evaluate the long-term signal retention characteristics of the encapsulated substrates in live cells.

## 2. Results

Since the orthogonality of the given substrates-enzymes combination is the key in this study, we initially conducted a preliminary experiment for the confirmation of the specificity of the given enzyme reactions utilizing a mixture of α- and β-substrates with distinct fluorophores (Figure S1). Also, the enzyme specificities utilizing α- and β-fluorogenic substrates have been well documented in previous studies [3,4,5].

### 2.1. Encapsulation of 4MUG/4FMUG in GlcCer Liposomes and Their Stability

GlcCer-based liposomes were prepared by the thin-film hydration method using GlcCer obtained from rice bran and dipalmitoylphosphatidylglycerol (DPPG-Na) (Figure 1B), followed by extrusion through 100 nm polycarbonate membranes. A small portion of DPPG-Na was added in order to provide a surface-negative charge to avoid aggregation of liposomes [29]. Dynamic light scattering (DLS) analysis and polydispersity index (PDI) showed that the liposomes had a mean hydrodynamic diameter of approximately 91–115 nm (Table 1). Encapsulation of α-4MUG, β-4MUG, or β-4FMUG within the aqueous lumen did not significantly affect the particle size. The encapsulation efficiency for both α- and β-substrates, determined by acid hydrolysis followed by fluorescence quantification of the released 4MU (approximately 8%), was consistent with passive loading into the internal aqueous phase [30]. Free, unencapsulated substrates were removed by dialysis against PBS using a 2 kDa MWCO dialysis cassette.

To assess the stability of the substrate-loaded liposomes under physiological conditions, dialyzed liposomes were incubated in a cell culture incubator (37 °C, 5% CO_2_) for up to 8 h. An aliquot was incubated with exogenous α- or β-glucosidase for 3 min, immediately followed by heat inactivation in an 80 °C-oil bath for 3 min. Fluorescence measurement clearly showed no leakage over incubation time, yet the liposomes contain encapsulated substrates, as confirmed by sonication to disrupt the vesicles (Figure S2). This indicates a very low likelihood of significant substrate leakage from intact liposomes to the surrounding medium and thus the stability of the liposomes.

### 2.2. Early Intracellular Events Leading to Hydrolysis of α- and β-MUG in Undifferentiated PC12 Cells

**Figure 2:**
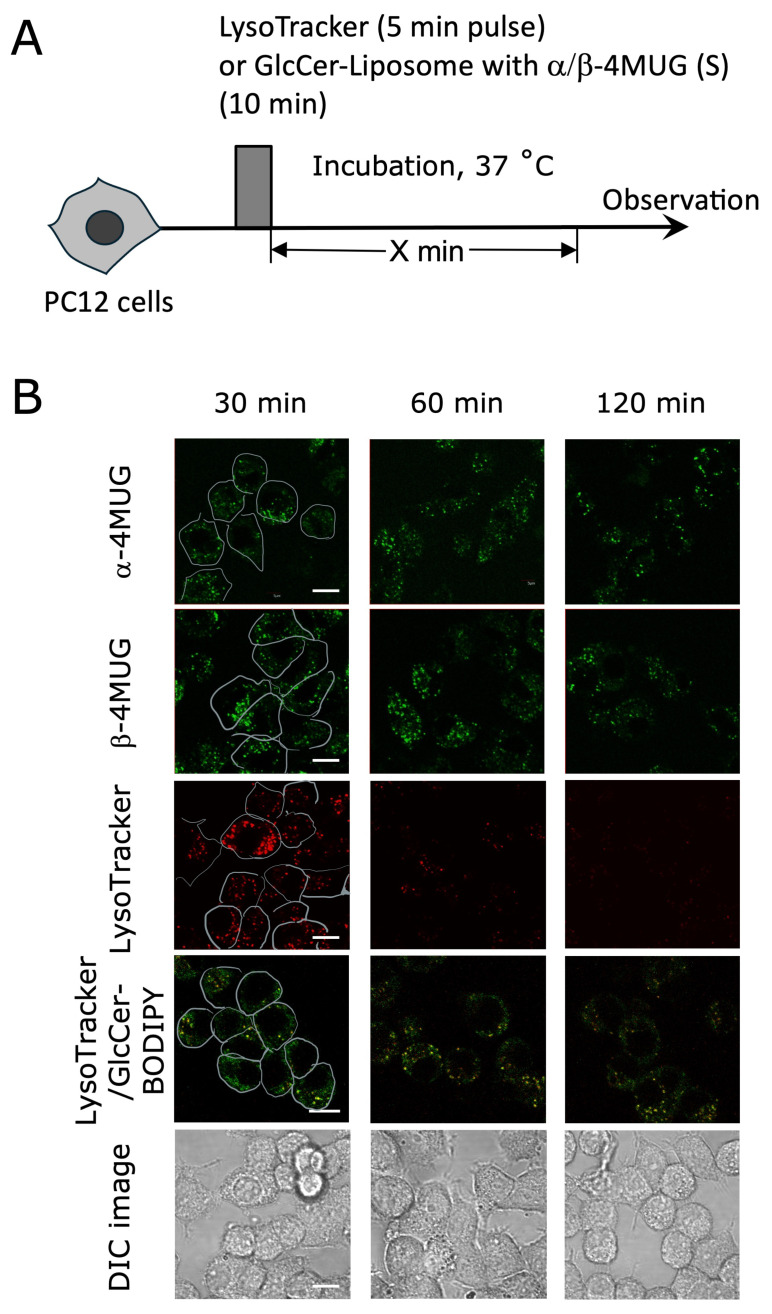
Early intracellular fluorescence after pulse exposure to GlcCer liposomes encapsulating α-4MUG or β-4MUG in undifferentiated PC12 cells. (**A**) Experimental protocol. PC12 cells were pulse-treated with GlcCer-liposomes encapsulating α-4MUG/β-4MUG (10 min) or LysoTracker (5 min). Cells were then incubated at 37 °C and imaged at 30, 60, and 120 min. (**B**) Confocal images at 30, 60, and 120 min after the pulse treatment. First row: Cells treated with α-4MUG liposomes. Second row: Cells treated with β-4MUG liposomes. Third row: Images with LysoTracker. Fourth row: Co-staining images of the cells with liposomes consisting of GlcCer and BODIPY-labeled GlcCer (0.24%) and LysoTracker. DIC images in the bottom row were acquired from the LysoTracker condition and were shown as reference images. Cell outlines are indicated for the first column. In the α-4MUG and β-4MUG panels, the fluorescence signals are displayed in green for visibility, although the original signals are blue. In the LysoTracker panel, the signal is shown in red. In the merged LysoTracker/GlcCer-BODIPY panels, green indicates GlcCer-BODIPY and red indicates LysoTracker. DIC images are shown in grayscale. Scale bar: 10 μm.

To investigate the early intracellular processing of the encapsulated substrates, undifferentiated PC12 cells were incubated with GlcCer liposomes co-encapsulating α- and β-4MUG for 10 min (pulse introduction) (Figure 2A). After gentle washing with PBS, cells were imaged in phenol red-free, low-glucose DMEM within 120 min. Confocal images showed the intense green dots indicating hydrolysis of both α- and β-linked substrates in vesicular organelles over 120 min, where pulse-introduced LysoTracker faded gradually over the given time (Figure 2B). Fluorescence became detectable after 30 min, suggesting that liposome uptake precedes detectable substrate hydrolysis. In contrast, co-localization of GlcCer liposomes containing BODIPY C5-labeled glucosyl sphingosine (GlcCerBODIPY) with LysoTracker revealed abundant fluorescence in the cytoplasm, along with punctate structures co-localizing with LysoTracker (Figure 2B, fourth row). Further analysis indicates BODIPY originated fluorescence partially co-localized with that of LysoTracker, which is also indicated by Pearson’s co-efficiency and Mander’s tM values (Figure S3).

### 2.3. Differential Analysis of α- and β-Glucosidase Activities in Live PC12 Cells

It has been known that α- and β-glucosidases are located at lysosomes, yet relative activities of these enzymes inside the cell have not been reported. To enable simultaneous visualization of both α- and β-glucosidase activities, a combination of α-4MUG and β-4FMUG was chosen. Upon action of individual enzymes, the distinguishable fluorophores, namely 4MU (ex. 380 nm, em. 454 nm) and 4FMU (ex. 385 nm, em. 502 nm), are released (Figure 1C). For microscopic observations, fluorophores can be sequentially excited at 405 and 488 nm while a set of cut-off filters (BP 450/50 and 525/50) was used for the detection of individual fluorescence signals.

**Figure 3:**
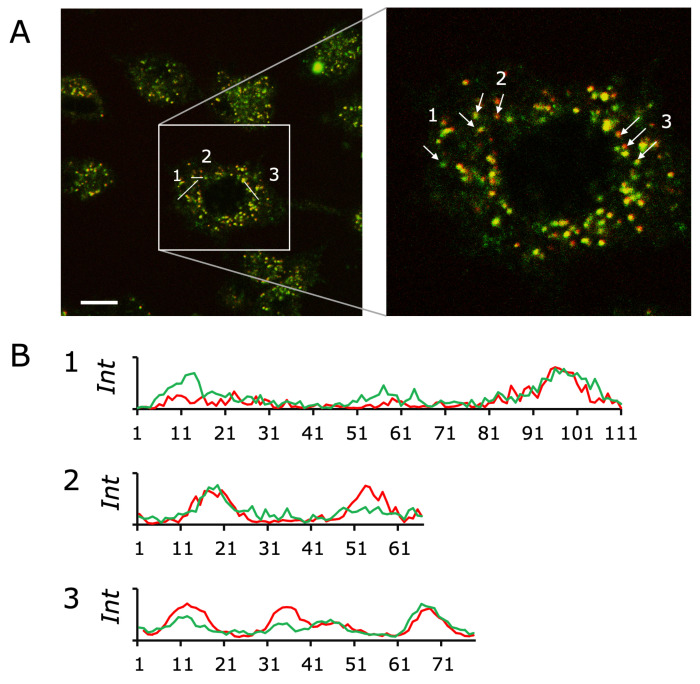
Differential fluorescence signals of 4MU and 4FMU after delivery of α-4MUG and β-4FMUG by GlcCer-liposomes in undifferentiated PC12 cells. (**A**) Representative confocal image acquired 30 min after a pulse introduction of GlcCer liposomes encapsulating α-4MUG and β-4FMUG. Red: 4MU signals (for visibility); green: 4FMU signals. The right image is a magnified view of the boxed region in the left image. The arrows and labels (1–3) indicate the positions used for line-scan analysis. Scale bar: 10 μm. (**B**) Line-scan intensity profiles extracted along positions 1–3 in panel (**A**). Red trace: 4MU; Green trace: 4FMU. The *y*-axis indicates relative fluorescence intensity and the *x*-axis is the pixel counts.

GlcCer-liposomes encapsulating both α-4MUG and β-4FMUG were pulse-introduced into cultured PC12 cells as described in Section 2.2, and localizations of fluorescence were obtained after 30 min (Figure 3A), where punctate intracellular fluorescence signals were observed. For visibility, the 4MU signal was displayed in red and the 4FMU signal in green, and individual puncta exhibited variable red/green composition. We then extracted line-scan intensity profiles along the three representative lines (1–3) indicated in Figure 3A (Figure 3B). The red and green traces showed peaks with different relative amplitudes and degrees of overlap across the three regions, indicating compartment-to-compartment variability in the two fluorescence signals.

### 2.4. Long-Term Retention and Slow Release of Encapsulated Substrates

To evaluate the long-term behavior of the encapsulated substrates, PC12 cells were incubated after pulse exposure of GlcCer-liposomes (10 min) loaded with α-4MUG/β-4FMUG and imaged for up to 148 h (even after cells were divided several times) (Figure S4). At 24 h, the overall intensity of both fluorescent signals was markedly reduced compared to the 30–60 min time frame, yet weaker signals were still detectable in many cells. The residual fluorescence primarily manifested as small punctate structures, indicating that a fraction of the encapsulated substrates or their products persisted within intracellular compartments. After 148 h, both red and green signals were observable, suggesting that individual GlcCer liposomes can retain and slowly release substrates over an extended timescale.

## 3. Discussion

This study establishes a glucosylceramide-based liposomal system capable of co-encapsulating α-4MUG/β-4MUG and a fluorinated β-4MUG analogue, β-4FMUG, for monitoring relative glycosidase activities in live PC12 cells over time (Figure 1A,C). Our work yields several key conclusions. First, GlcCer liposomes with a diameter of approximately 110 nm can encapsulate the substrates with similar efficiency (Table 1). The Glc-ase treatment of the liposome-containing assay medium resulted in negligible leakage of the encapsulated substrates. Their retention inside the liposomes was confirmed by fluorescence generated upon enzymatic hydrolysis after liposome disruption (Figure S2).

Second, upon uptake by PC12 cells, both α- and β-substrates start to be hydrolyzed within tens of minutes, generating a fluorescent signal detectable without cell lysis (Figure 2). GlcCer-rich liposomes are expected to undergo time-dependent changes within the endocytic and lysosomal pathway. The neutral nature and hydrogen-bonding capability of the headgroup of GlcCer may promote tight bilayer packing [18], which could support prolonged retention of encapsulated cargo. These results indicate that the incorporated liposomes were stored in possibly endosomes and, over time, fused with lysosomes for the enzymatic hydrolysis. However, in our imaging experiments, fluorescent products became detectable within tens of minutes after the pulse introduction, suggesting that α- and β-Glc-ase can access a fraction of the cargo after the fusion of endosomes and lysosomes without requiring complete vesicle disintegration.

Third, the observed locations of fluorescent molecules produced from α-4MUG upon actions of corresponding enzymes are highly correlated with lysosome-like punctate structures (Figure 2 and Figure S3). The observed punctate was confirmed to be lysosomes using BODIPY-tagged GlcCer in the GlcCer liposomes without encapsulating substrates in the co-localization experiments with LysoTracker (Figure 2B, fourth row). The BODIPY-based fluorescence (shown in green) partially overlapped with LysoTracker (red), where faint green color was scattered in the cell, indicating GlcCer was present in endosomes after the internalization event. Finally, signals of both 4MU and 4FMU released from respective α- and β-substrates persisted for a longer duration as compared with pulse-introduced LysoTracker (Figure 2B).

Our results highlight the potential of GlcCer-containing liposomes as low-leakage carriers for fluorogenic enzyme substrates. Traditional fluorescence assays are typically performed on lysed samples after cell culture [31], and their application in live cells is limited by poor cellular permeability and pH-dependent fluorescence of the products [32]. By encapsulating α-4MUG and β-4FMUG within GlcCer liposomes and removing free substrate via dialysis, we effectively restricted the exposure of substrates to lysosomal glycosidases. The GlcCer bilayer provides a relatively rigid barrier that stabilizes the encapsulated substrates in the culture medium, aligning with previous reports that GlcCer-rich liposomes can form ordered phases and modulate cellular lipid metabolism [18]. The lysosomal localization of α-4MUG-derived 4MU and β-4FMUG-derived 4FMU is consistent with the previous reports regarding localizations of α- and β-glucosidases [23,33]. The spatial patterns of α- versus β-substrate hydrolysis provide insight into compartmentalized glycosidase activity. In Figure 3, 4MU (red) and 4FMU (green) signals appeared as punctate structures, and the red/green balance varied among puncta. Line-scan intensity profiles across three representative regions (Figure 3B) further showed differences in peak ratios and degrees of overlap between the two observation channels, indicating heterogeneity in the relative contributions of α- and β-substrate hydrolysis at the single-compartment level.

The detection of a fluorescent signal up to 148 h post-loading indicates that GlcCer-liposomes and/or their associated lipids/substrates are cleared from PC12 cells relatively slowly. This phenomenon is consistent with prior observations that liposomes can maintain intracellular endosomal/lysosomal distribution and extended retention [34], and with the knowledge that glycosphingolipids undergo endocytic recycling and salvage pathways, resulting in overall turnover half-lives on the scale of days [35]. Consequently, GlcCer-liposomes hold potential as carriers for sustained delivery and long-term tracking of slower metabolic processes. The prolonged retention of 4MU and 4FMU signals suggests that internalized liposomes and/or their associated substrates or products may persist in endosomal/lysosomal compartments, resulting in slow fluorescence release over time. This property may be relevant to future drug-delivery applications. This was clearly supported by co-localization experiments using GlcCer liposomes containing BODIPY-C5–labeled glucosyl ceramide and LysoTracker (Figure 2B, fourth row). Immediately after internalization, a broadly distributed, non-punctate fluorescence pattern was observed in the cytoplasm. Over time, however, punctate structures showing co-localization of the liposomes with LysoTracker became increasingly prominent. These observations suggest that endosomes subsequently fuse with lysosomes, where lysosomal enzymes hydrolyze substrates encapsulated within the liposomes following disruption of the liposomal membrane.

Another advantage of this system is its capacity for the simultaneous delivery and monitoring of multiple substrates. By combining the α-4MUG with β-4FMUG, we distinguished between α- and β-substrate hydrolysis within the same cell without significant spectral crosstalk. This multiplexing capability could be extended to other combinations of 4MU-based substrates and their analogues targeting different glycosidases, enabling comparative analysis of multiple enzyme activities under various physiological and pathological conditions.

The current experimental setup does not provide quantitative information, thus it may require complemented biochemical experiments in future investigations. Despite the limitation, our results demonstrate that GlcCer-liposomes co-encapsulating α-4MUG and β-4FMUG provide a practical platform for imaging relative glycosidase activities in live cells. This approach is particularly suited for dissecting the roles of lysosome-associated glycosidases in diseases.

## 4. Materials and Methods

### 4.1. Reagents

α-4-Methylumbelliferyl glucopyranoside (α-4MUG) and β-4-methylumbelliferyl glucopyranoside (β-4MUG) were purchased from Funakoshi Co., Ltd. (Tokyo, Japan) and Santa Cruz Biotechnology, Inc. (Dallas, TX, USA), respectively. All other chemicals and solvents were of analytical or HPLC grade. Glucosylceramide (GlcCer) was purified from rice bran-derived glycosphingolipids (OKAYASU, Saitama, Japan). PC12 cells (rat pheochromocytoma; RIKEN BRC Cell Bank, Tsukuba, Japan) were cultured under standard conditions (37 °C, 5% CO_2_).

### 4.2. Preparation of GlcCer Liposomes Encapsulating 4MUG/4FMUG

GlcCer (6.5 mg, 8.2 µmol) and DPPG-Na (0.3 mg, 0.4 µmol) were dissolved in chloroform/methanol (2:1, *v*/*v*, 1 mL). The solvent was evaporated using a rotary evaporator (EYELA, Tokyo, Japan), and the resulting film was dried under vacuum for 3 h. The film was hydrated with PBS (2 mL) containing α-4MUG, β-4MUG (each 0.35 mg, 1.03 μmol), and/or β-4FMUG (0.40 mg, 1.03 μmol) at 60 °C with intermittent vortex mixing. The resulting multilamellar vesicle suspension was extruded through a 100 nm-mesh polycarbonate membrane under 0.8 MPa nitrogen pressure for 10 cycles to obtain liposomes. For co-encapsulation experiments, both α-4MUG and β-4FMUG were dissolved in PBS at a concentration of 0.5 mM. The free substrates in the solution were removed by dialysis against PBS using a dialysis cassette with a 2 kDa molecular weight cutoff (MWCO). The dialysis was performed at 4 °C for 16 h with several changes in the PBS buffer.

### 4.3. Particle Size and Encapsulation Efficiency

Particle size distribution was determined at 25 °C using a Zetasizer Nano (Malvern Instruments Ltd., Worcestershire, UK) in DLS mode. To determine encapsulation efficiency, an aliquot of dialyzed liposomes was mixed with two volumes of ethanol and vortexed at 70 °C for 3 min to fully release the encapsulated substrates. It was then incubated with 0.5 M HCl at 100 °C for 5 h to hydrolyze the fluorogenic substrates. After neutralization, the fluorescence of 4MU was measured using a microplate reader (SH-9000Labo, CORONA ELECTRIC, Ibaraki, Japan) with excitation at 405 nm. Encapsulation efficiency was calculated as the ratio of encapsulated substrate amount to the initially added total amount.

### 4.4. In Vitro Leakage Assay

To assess substrate leakage under standard conditions, dialyzed liposomes were placed in a cell culture medium and incubated (37 °C, 5% CO_2_) for up to 8 h. An aliquot was then incubated with exogenous α- or β-glucosidase for 3 min, immediately followed by heat inactivation in an 80 °C-oil bath for 3 min before fluorescence measurement. In parallel, an aliquot of the same liposome batch was sonicated for 5 min during the incubation period to disrupt the vesicles, followed by the same enzyme treatment and heat inactivation.

### 4.5. Cell Culture

PC12 cells were cultured in low-glucose (1000 mg/L) DMEM supplemented with 10% fetal bovine serum, 10% horse serum, and 1% penicillin-streptomycin at 37 °C under 5% CO_2_.

### 4.6. Liposome Incorporation and Confocal Imaging of the Cell

For imaging experiments, undifferentiated PC12 cells were seeded in glass-bottom dishes and cultured overnight. GlcCer liposomes co-encapsulating 4MUG/4FMUG were added at a final concentration of 0.5 mM GlcCer (formulated in liposomes) and incubated with the cells for 10 min. After incubation, the medium was removed, and the cells were gently washed twice with PBS. Live-cell imaging was performed directly in phenol red-free medium. Fluorescence images were acquired on a confocal laser scanning microscope (FV-1000, Olympus or A1 plus confocal, Tokyo, Japan, Nikon, Tokyo, Japan) equipped with a 60× water-immersion or 100× oil-immersion objective lens. The fluorescence signal from 4MU was excited at 405 nm and detected using a 450/50 nm-band-pass filter, while that from β-4FMUG was excited at 488 nm and detected (band-pass filter: 525/50 nm).

### 4.7. Image Analysis

Image analysis was performed using Fiji (ImageJ; version 1.54p) and NIS-Elements AR software (version 4.51.00, Nikon, Tokyo, Japan). Linear intensity profiles were drawn across selected regions of interest to assess the co-localization of “red” and “green” signals within individual fluorescent puncta.

## 5. Conclusions

This study developed a glucosylceramide (GlcCer)-enriched liposomal platform for the in situ multiplexed visualization of glycosidase activities in live PC12 cells. The fluorogenic substrates, α- and β-linked 4-methylumbelliferyl glucosides, were delivered to lysosomes, as confirmed by distinct fluorescence signals. Furthermore, fluorescence corresponding to individual enzymatic reactions was detectable for up to 148 h. The observed long-term retention was attributed to endosomal storage.

Given the clinical importance of α- and β-glucosidases, this platform may provide a useful basis for future diagnostic and therapeutic approaches targeting diseases associated with lysosomal glucosidase disorders.

## Figures and Tables

**Table 1:** Physical properties of the liposomes.

		Size (nm)	PDI ^1^	Zeta Potential ^2^ (mV)
GlcCer + DPPG	with α-4MUG	91.3 ± 0.6, *n* = 3	0.182 ± 0.002, *n* = 3	−14.7 ± 2.3, *n* = 5
	with β-4FMUG	105.9 ± 1.1, *n* = 3	0.182 ± 0.011, *n* = 3	−12.2 ± 2.8, *n* = 5
	+GlcCerBODIPY ^3^	114.9 ± 1.2, *n* = 3	0.150 ± 0.023, *n* = 3	−12.1 ± 1.4, *n* = 5
DOPC + Colesterol ^4^		111.7 ± 3.8, *n* = 3	0.144 ± 0.009, *n* = 3	−1.64 ± 1.34, *n* = 5

^1^ PDI: polydispersity index. ^2^ Zeta potentials were measured in 10 mM PIPES (pH 7.4) containing 150 mM NaCl. ^3^ The GlcCerBODIPY-containing liposome was prepared at a molar ratio of GlcCer:DPPG-Na:GlcCerBODIPY = 20:1:0.05. ^4^ A standard liposome consisting of DOPC and cholesterol at a 1:1 molar ratio.

## Data Availability

The original contributions presented in this study are included in the article/Supplementary Materials. Further inquiries can be directed to the corresponding author.

## Supplementary Materials

The following supporting information can be downloaded at: https://www.mdpi.com/article/10.3390/ijms27062749/s1.

## Abbreviations

The following abbreviations are used in this manuscript:

4FMUG	4-(trifluoromethyl)umbelliferyl glucoside
4MU	4-Methylumbelliferone
DIC	Differential interference contrast
DLS	Dynamic light scattering
DMEM	Dulbecco’s modified Eagle’s medium
ER	Endoplasmic reticulum
GCase	Glucocerebrosidase
GlcCer	Glucosylceramide
MWCO	Molecular weight cut-off
PBS	Phosphate-buffered saline
PDI	Polydispersity index

## References

[1] Futerman A.H., Van Meer G. (2004). The Cell Biology of Lysosomal Storage Disorders. Nat. Rev. Mol. Cell Biol..

[2] Helenius A., Aebi M. (2004). Roles of N-Linked Glycans in the Endoplasmic Reticulum. Annu. Rev. Biochem..

[3] Gorantla J.N., Maniganda S., Pengthaisong S., Ngiwsara L., Sawangareetrakul P., Chokchaisiri S., Kittakoop P., Svasti J., Ketudat Cairns J.R. (2021). Chemoenzymatic and Protecting-Group-Free Synthesis of 1,4- Substituted 1,2,3-Triazole-α-D-glucosides with Potent Inhibitory Activity toward Lysosomal α-Glucosidase. ACS Omega.

[4] Gegg M.E., Burke D., Heales S.J.R., Cooper J.M., Hardy J., Wood N., Schapira A.H.V. (2012). Glucocerebrosidase Deficiency in Substantia Nigra of Parkinson Disease Brains. Ann. Neurol..

[5] Karpova E.A., Voznyi Y.V., Dudukina T.V., Tsvetkova I.V. (1991). 4-Trifluoromethylumbelliferyl Glycosides as New Substrates for Revealing Diseases Connected with Hereditary Deficiency of Lysosome Glycosidases. Biochem. Int..

[6] Dardis A., Michelakakis H., Rozenfeld P., Fumic K., Wagner J., Pavan E., Fuller M., Revel-Vilk S., Hughes D., Cox T. (2022). Patient Centered Guidelines for the Laboratory Diagnosis of Gaucher Disease Type 1. Orphanet J. Rare Dis..

[7] Oftedal L., Maple-Grødem J., Førland M.G.G., Alves G., Lange J. (2020). Validation and Assessment of Preanalytical Factors of a Fluorometric In Vitro Assay for Glucocerebrosidase Activity in Human Cerebrospinal Fluid. Sci. Rep..

[8] Yu C., Sun Q., Zhou H. (2013). Enzymatic Screening and Diagnosis of Lysosomal Storage Diseases. N. Am. J. Med. Sci..

[9] Wu X., Wang R., Kwon N., Ma H., Yoon J. (2022). Activatable Fluorescent Probes for in Situ Imaging of Enzymes. Chem. Soc. Rev..

[10] Zylberberg C., Matosevic S. (2016). Pharmaceutical Liposomal Drug Delivery: A Review of New Delivery Systems and a Look at the Regulatory Landscape. Drug Deliv..

[11] Reza S., Ugorski M., Suchański J. (2021). Glucosylceramide and Galactosylceramide, Small Glycosphingolipids with Significant Impact on Health and Disease. Glycobiology.

[12] Yamaguchi R., Kanie Y., Kazamaki T., Kanie O., Shimizu Y. (2023). Cellular Uptake of Liposome Consisting Mainly of Glucocerebroside from the Starfish *Asterias amurensis* into Caco-2 Cells. Carbohydr. Res..

[13] Tokudome Y., Endo M., Hashimoto F. (2014). Application of Glucosylceramide-Based Liposomes Increased the Ceramide Content in a Three-Dimensional Cultured Skin Epidermis. Skin. Pharmacol. Physiol..

[14] Greene L.A., Tischler A.S. (1976). Establishment of a Noradrenergic Clonal Line of Rat Adrenal Pheochromocytoma Cells Which Respond to Nerve Growth Factor. Proc. Natl. Acad. Sci. USA.

[15] Mutoh T., Tokuda A., Inokuchi J., Kuriyama M. (1998). Glucosylceramide Synthase Inhibitor Inhibits the Action of Nerve Growth Factor in PC12 Cells. J. Biol. Chem..

[16] Crespo P.M., Silvestre D.C., Gil G.A., Maccioni H.J.F., Daniotti J.L., Caputto B.L. (2008). C-Fos Activates Glucosylceramide Synthase and Glycolipid Synthesis in PC12 Cells. J. Biol. Chem..

[17] Brailoiu E., Churamani D., Pandey V., Brailoiu G.C., Tuluc F., Patel S., Dun N.J. (2006). Messenger-Specific Role for Nicotinic Acid Adenine Dinucleotide Phosphate in Neuronal Differentiation. J. Biol. Chem..

[18] Varela A.R.P., Couto A.S., Fedorov A., Futerman A.H., Prieto M., Silva L.C. (2016). Glucosylceramide Reorganizes Cholesterol-Containing Domains in a Fluid Phospholipid Membrane. Biophys. J..

[19] Yi X., Gao S., Gao X., Zhang X., Xia G., Liu Z., Shi H., Shen X. (2023). Glycolipids Improve the Stability of Liposomes: The perspective of Bilayer Membrane Structure. Food Chem..

[20] Maggio B., Ariga T., Sturtevant J.M., Yu R.K. (1985). Thermotropic Behavior of Glycosphingolipids in Aqueous Dispersions. Biochemistry.

[21] van Lummel M., van Blitterswijk W.J., Vink S.R., Jan Veldman R., van der Valk M.A., Schipper D., Dicheva B.M., Eggermont A.M.M., ten Hagen T.L.M., Verheij M. (2011). Enriching Lipid Nanovesicles with Short-chain Glucosylceramide Improves Doxorubicin Delivery and Efficacy in Solid Tumors. FASEB J..

[22] Roig-Zamboni V., Cobucci-Ponzano B., Iacono R., Ferrara M.C., Germany S., Bourne Y., Parenti G., Moracci M., Sulzenbacher G. (2017). Structure of Human Lysosomal Acid α-Glucosidase–A Guide for the Treatment of Pompe Disease. Nat. Commun..

[23] Moreland R.J., Jin X., Zhang X.K., Decker R.W., Albee K.L., Lee K.L., Cauthron R.D., Brewer K., Edmunds T., Canfield W.M. (2005). Lysosomal Acid Alpha-Glucosidase Consists of Four Different Peptides Processed from a Single Chain Precursor. J. Biol. Chem..

[24] Grabowski G.A., Gatt S., Kruse J., Desnick R.J. (1984). Human Lysosomal Beta-Glucosidase: Kinetic Characterization of the Catalytic, Aglycon, and Hydrophobic Binding Sites. Arch. Biochem. Biophys..

[25] Sawkar A.R., Cheng W.-C., Beutler E., Wong C.-H., Balch W.E., Kelly J.W. (2002). Chemical Chaperones Increase the Cellular Activity of N370S β-Glucosidase: A Therapeutic Strategy for Gaucher Disease. Proc. Natl. Acad. Sci. USA.

[26] Fu Q., Zhao Y., Yang Z., Yue Q., Xiao W., Chen Y., Yang Y., Guo L., Wu Y. (2019). Liposomes Actively Recognizing the Glucose Transporter GLUT1 and Integrin αvβ3 for Dual-targeting of Glioma. Arch. Pharm..

[27] Marsh C.A., Levvy G.A. (1956). Synthesis of 4-Methylumbelliferone β-D-Glucuronide, A Substrate for the Fluorimetric Assay of β-Glucuronidase. Nature.

[28] Hong J., Cao J., Chen X., Yang S., Zhang B., Tang R., Huang Y., Gan N., Huang S. (2025). A Novel Electrochemical Strategy for Rapid and Sensitive α-Glucosidase Activity Detection and Inhibitor Discovery Based on Distinct Oxidation Potentials of Substrate and Product. Microchem. J..

[29] Lombardo D., Kiselev M.A. (2022). Methods of Liposomes Preparation: Formation and Control Factors of Versatile Nanocarriers for Biomedical and Nanomedicine Application. Pharmaceutics.

[30] Wang Y., Tu S., Pinchuk A.N., Xiong M.P. (2013). Active Drug Encapsulation and Release Kinetics from Hydrogel-in-Liposome Nanoparticles. J. Colloid. Interface Sci..

[31] Harlan F.K., Lusk J.S., Mohr B.M., Guzikowski A.P., Batchelor R.H., Jiang Y., Naleway J.J. (2016). Fluorogenic Substrates for Visualizing Acidic Organelle Enzyme Activities. PLoS ONE.

[32] Profeta G.S., Pereira J.A.S., Costa S.G., Azambuja P., Garcia E.S., Moraes C.S., Genta F.A. (2017). Standardization of a Continuous Assay for Glycosidases and Its Use for Screening Insect Gut Samples at Individual and Populational Levels. Front. Physiol..

[33] Reczek D., Schwake M., Schröder J., Hughes H., Blanz J., Jin X., Brondyk W., Van Patten S., Edmunds T., Saftig P. (2007). LIMP-2 Is a Receptor for Lysosomal Mannose-6-Phosphate-Independent Targeting of β-Glucocerebrosidase. Cell.

[34] Guenoun J., Koning G.A., Doeswijk G., Bosman L., Wielopolski P.A., Krestin G.P., Bernsen M.R. (2012). Cationic Gd-DTPA Liposomes for Highly Efficient Labeling of Mesenchymal Stem Cells and Cell Tracking with MRI. Cell Transplant..

[35] Tettamanti G. (2004). Ganglioside/Glycosphingolipid Turnover: New Concepts. Glycoconj. J..

