# Supplementary material for: In Situ Differential Analysis of α- and β-Glycosidase Activities in Lysosomes After Internalization Using Glucosylcerebroside-Based Liposomes
Source: Int J Mol Sci. 2026 Mar 18;27(6):2749. doi: 10.3390/ijms27062749 (PMC13027016; doi:10.3390/ijms27062749)
Supplement: Supplementary file 1 [file ijms-27-02749-s001.zip › ijms-4176117-supplementary.pdf]

Supplementary Materials

In situ Differential Analysis of α- and β-Glycosidase Activities in Lysosomes after Internalization using Glucosylcerebroside-based Liposomes

Yi Wei[1] and Osamu Kanie[1,2,3]

[1]Graduate School of Science and Technology, [2]Department of Bioengineering, and [3]Micro/Nano Technology Center, Tokai University, Hiratsuka, Kanagawa 259-1292, Japan

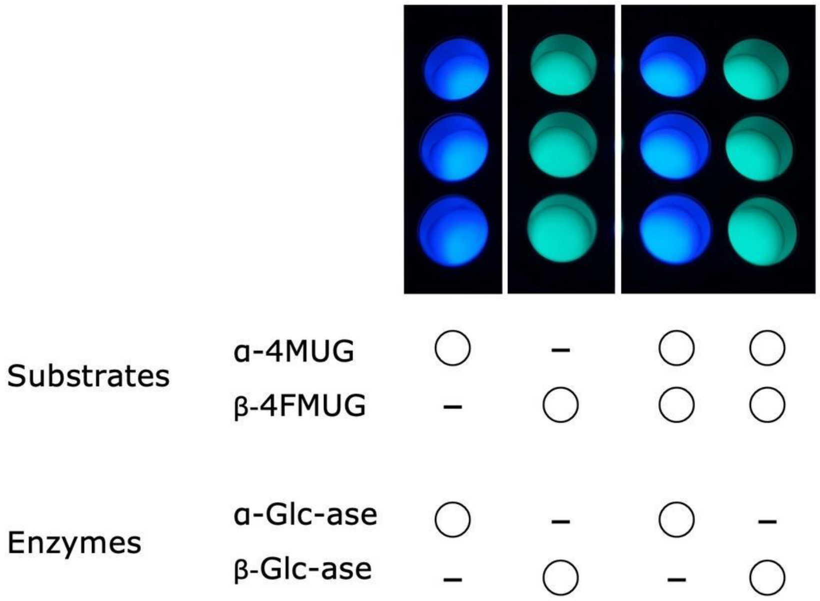

Figure S1. Substrate specificities of α-/β-Glc-ases.

For successful differential analysis of α-/β-Glc-ases, the orthogonality of a set of enzyme reactions in combination of α-4MUG and β-4FMUG has to be confirmed. To ascertain the specificities of these enzymes, a mixture of α-4MUG and β-4FMUG was separately treated with α-Glc-ase (from brewer's yeast) and β-Glc-ase (from almonds).

The result shows a complete selectivity of α- and β-Glc-ase reactions for a mixture of α-4MUG and β-4FMUG.

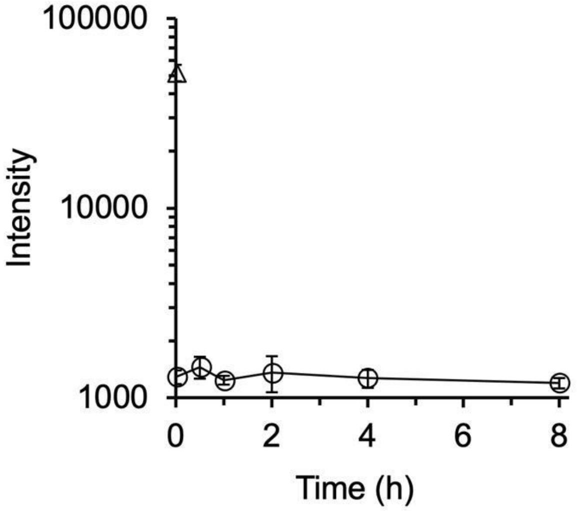

Figure S2. Stability of substrate-loaded GlcCer-liposomes. GlcCer-liposomes encapsulating α-4MUG were incubated in cell culture medium at 37 °C. At the indicated times, aliquots were collected and treated with α-glucosidase, with or without prior sonication. After heat inactivation of the enzyme (80 °C, 3 min), fluorescence intensities were measured. Open circles indicate fluorescence detected without sonication, and open triangles indicate fluorescence detected after sonication. Data were shown as mean ± SD (*n = 3*).

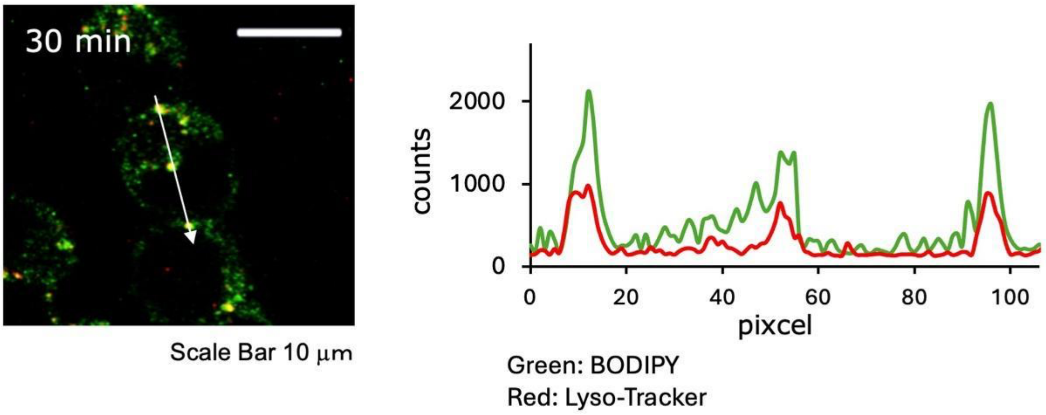

Pearson's R value: 0.44±0.052 (n = 4)
Manders' tM1: 0.44±0.034 (n = 4)
Manders' tM2: 0.51±0.033 (n = 4)

Figure S3. Localization of GlcCer-liposomes.
GlcCer-liposomes containing GlcCerBODIPY (0.24%) were rapidly taken up by cultured PC12 cells within 5 min (image not shown) and subsequently trafficked to lysosomes, as indicated in Fig. 2. The fusion process is evident in the representative image obtained 30 min after pulse exposure (upper panel). Cross-sectional analysis reveals partial overlap of the two fluorescence signals. Green fluorescence detected in cytosolic regions lacking LysoTracker signals is attributed to endosomes. This interpretation is supported by the stronger green fluorescence observed in the cytosol at the early stage. Statistical analysis of fluorescence intensities at the pixel level within the region of interest further supports the notion that GlcCer-liposomes are first localized in endosomes and subsequently fuse with lysosomes.

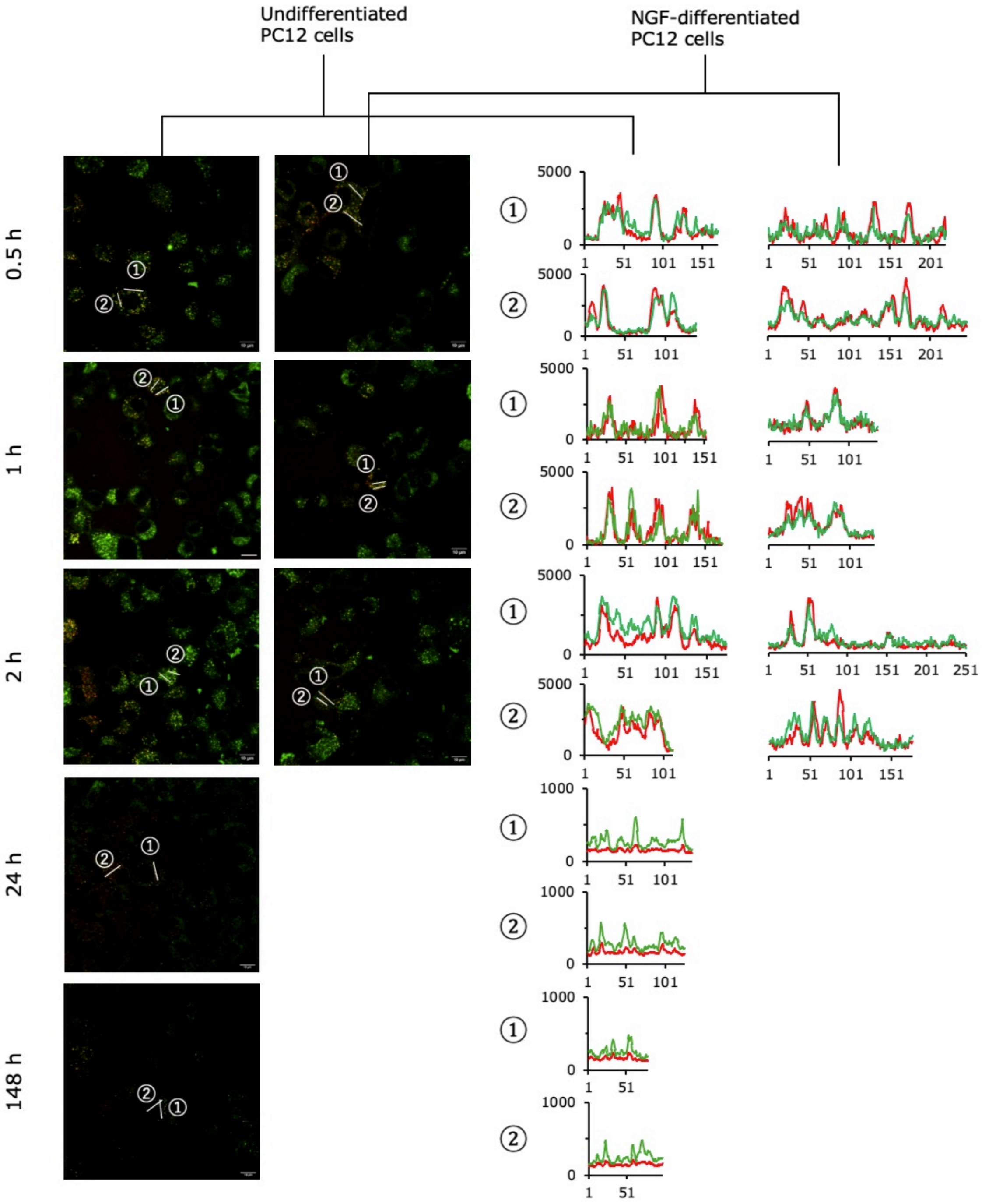

Figure S4. Time-course confocal images and their analyses after pulse exposure of GlcCer-liposomes co-encapsulating *α*-4MUG and β-4FMUG in undifferentiated and NGF-differentiated PC12 cells.

It is important to examine the long-term effect of the substrate-encapsulating liposomes inside the cells when considered the future application with a pharmaceutical drugs. For this purpose, undifferentiated PC12 cells (first column) and NGF-differentiated PC12 cells (second column) were pulse-treated with GlcCer-liposomes co-encapsulating α-4MUG and β-4FMUG and imaged over time.
Images at 0.5 h, 1 h, and 2 h are shown for both cell conditions. Additional late time points (24 h and 148 h) are shown for undifferentiated PC12 cells (first column). The images display the 4MU channel (shown in red) and the 4FMU channel (green). Scale bars: 10 μm.
